# The Minimum Number of Ablation Lines for Complete Isolation of the Pulmonary Veins during Thoracoscopic Ablation for Atrial Fibrillation

**DOI:** 10.3390/life13030770

**Published:** 2023-03-13

**Authors:** Min Suk Choi, Yoonseo Lee, Dong Seop Jeong

**Affiliations:** 1 Department of Thoracic and Cardiovascular Surgery, Dongguk University Ilsan Hospital, Dongguk University College of Medicine, Goyang-si 10326, Republic of Korea; 2 Department of Thoracic and Cardiovascular Surgery, Samsung Medical Center, Sungkyunkwan University School of Medicine, Seoul 06351, Republic of Korea

**Keywords:** atrial fibrillation, entrance block, exit block, pulmonary vein isolation, totally thoracoscopic ablation

## Abstract

Total thoracoscopic ablation has been recommended as a class IIa indication for atrial fibrillation. However, the optimal number of ablation lines for pulmonary vein isolation has not yet been proposed. This study aimed to report the minimum number of ablation lines required to achieve an intraoperative conduction block. This study included a total of 20 patients who underwent total thoracoscopic ablation from December 2020 to July 2021. The epicardial conduction block was checked after each ablation line of pulmonary vein antral clamping. The median age was 61 years old. The median duration of atrial fibrillation since the first diagnosis was 78 months. Pulmonary vein isolation with bidirectional conduction block was confirmed in 90% of patients. A median of six ablation lines around each pulmonary vein antrum were performed according to our protocol even after the conduction block was verified. The median number of ablations to achieve an exit block was two on the right side and 3.5 on the left side. We found that most conduction blocks were achieved within three ablations around the pulmonary vein antrum. Our results may provide evidence to reduce the number of unnecessary ablation lines in the future.

## 1. Introduction

Atrial fibrillation (AF) is the most common type of arrhythmia, and it carries significant risk of cardiac and cerebrovascular morbidities [1]. The treatments for converting AF to sinus rhythm include cardioversion, pharmacologic control, catheter ablation, and surgical treatment. Rhythm control with medication or pulmonary vein isolation with ablation is recommended for symptomatic AF [2,3]. Catheter ablation for AF is considered for drug refractory cases as a class I recommendation, and it is a class IIa recommendation for the first-line therapy [3]. The Cox-maze procedure, which is the typical surgical treatment, has been renowned for its long-term efficacy, but the invasiveness of the procedure has limited its indications [4,5,6]. Recently, total thoracoscopic ablation (TTA) has been widely accepted for its lesser invasiveness and satisfactory efficacy [7,8,9,10,11,12,13], and it is recommended as a class IIa indication for symptomatic and drug-refractory AF in selected patients [2,3,14].

Specific mechanisms and determinants of atrial fibrillation have not yet been identified. They are also recognized as being multifactorial and sharing properties of reentry, automaticity, and triggered activity. Rapid discharge from the pulmonary veins, the most common trigger of AF, may also play a perpetuating role, more so in paroxysmal AF than in persistent AF [15], which is why pulmonary vein isolation is particularly effective for eliminating AF. It is recognized as an integral part of the procedure to treat AF [16]. Therefore, pulmonary vein clamping lines are also very significant during TTA [14,16]. Surgeons create a certain number of ablation lines initially encircling the pulmonary veins and test the exit and entrance blocks. If a block is not achieved, additional lines are created. The preferred number of ablation lines ranges from two to 10 [17,18,19].

Although more ablation lines may guarantee a conduction block, procedure-related complications, such as pulmonary vein stenosis, may increase with more ablation lines. The rate of pulmonary vein stenosis after radiofrequency ablation is known to be related to the methods of ablation and techniques [20]. Therefore, although pulmonary vein stenosis after TTA has not been commonly reported, excessive ablation is likely to increase the probability of complications.

The primary endpoint of this study was to determine the minimum number of ablation lines required around the pulmonary vein antrum to achieve intraoperative conduction block during TTA.

## 2. Materials and Methods

### 2.1. Study Population

This was a single-center, retrospective observational study that was approved by the Institutional Review Board (IRB) of the medical center (IRB approval no. 2022 06 130 001; IRB approval date, 1 July 2022). The indication for TTA was mainly persistent or long-standing persistent AF and medically refractory paroxysmal AF. A total of 20 patients who underwent TTA in the center from December 2020 to July 2021 by one dedicated surgeon were included in this study.

### 2.2. Totally Thoracoscopic Ablation

**Figure 1:**
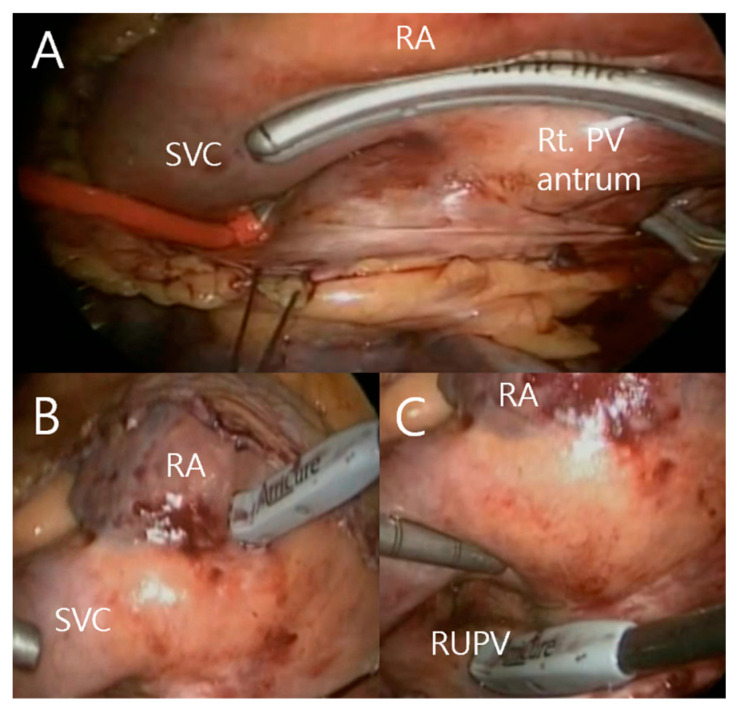
Right-side thoracoscopic view of pulmonary vein antral clamp ablation (**A**), entrance block test (**B**), and exit block test (**C**). SVC; superior vena cava, PV; pulmonary vein, RA; right atrium, Rt.; right, RUPV; right upper pulmonary vein.

The surgery is a video-assisted thoracoscopic surgical technique without the aid of mini-thoracotomy, the Da Vinci system, or cardiopulmonary bypass. It is conducted under general anesthesia, and the patient is intubated with a double-lumen endotracheal tube for selective one-lung ventilation. The central venous catheter is inserted through the femoral vein, not the internal jugular vein, because we sometimes ablate around the superior vena cava. Transesophageal echocardiography is performed in the operation room to verify the absence of a left atrial thrombus before the start of the operation and adequacy of the left atrial appendage excision at the end of the operation. The procedure is performed with the patient in the supine position. The procedure is performed using bilateral approaches, requiring only three ports per side: a 5-mm port introduced in the fourth intercostal space at the midaxillary line, a 5-mm port placed in the third intercostal space at the anterior axillary line, and a 12-mm port at the sixth intercostal space at the midaxillary line, respectively [21]. Starting on the right side, a 5-mm port is introduced in the fourth intercostal space at the midaxillary line. After carbon dioxide insufflation, usually at 8–10 mm Hg to expand the operative field and depress the diaphragm, the remaining two ports are placed in the third intercostal space at the anterior axillary line and the sixth intercostal space at the midaxillary line, respectively. The pericardium is incised from the superior vena cava to the inferior vena cava 2 or 3 cm anterior and parallel to the phrenic neve. Two tenting sutures at the posterior pericardial edge are brought through the skin and anchored. After pericardial tenting, dissection posterior to the inferior vena cava and inferior to the right inferior pulmonary vein is necessary to enter the oblique sinus. An angulated lighted dissector (AtriCure Lumitip Dissector, AtriCure, Inc., Cincinnati, OH, USA) is introduced into the chest through a port and passed into the oblique sinus inferior to the right lower pulmonary vein. While the superior vena cava is distracted medially and the right pulmonary artery is distracted superiorly, dissection posterior to the right upper pulmonary vein is completed with the lighted dissector. The dissector is exchanged for an 18Fr rubber band to secure the path. An AtriCure Isolator Transpolar Clamp (Atricure, Inc., Cincinnati, OH, USA), with its lower jaw connected with the end of the rubber band, is introduced through the port. The rubber band is then used to guide the lower jaw of the clamp behind the right pulmonary vein antrum as the upper jaw passes in front of the antrum. Correct positioning of the clamp on the pulmonary vein antrum and not on the pulmonary veins themselves is verified by directly inspecting the device after closing the jaws of the clamp. Pulmonary vein antrum isolation is performed by applying bipolar radiofrequency energy (Figure 1A). The temperature of the tissue 1.6 mm from the electrode is displayed on the generator [22]. A digital graph, located on the front panel of the generator, displays the conductance of the tissue clamped between the device jaws. When the conductance of the tissue decreases to less than 0.0025 siemens, typically after 8 s, an audible signal is automatically generated to indicate that the lesion is transmural. Ablation lines are visible on the epicardial surface.

Our protocol is to clamp six times around each pulmonary vein antrum. The box lesions, including the left atrial roof and floor lesions connecting both pulmonary veins, are created with an AtriCure linear pen device (Atricure, Inc., Cincinnati, OH, USA). Additional superior and inferior ablation lines connecting both pulmonary vein isolation lines are created epicardially using a linear pen device (AtriCure, Inc., Cincinnati, OH, USA). Ganglionated plexuses are subsequently ablated with bipolar radiofrequency energy with the aid of high-frequency stimulation. Confirmation of the ablation lines is obtained by pacing testing using the AtriCure Cooltip pen (Atricure, Inc., Cincinnati, OH, USA). The pericardium is closed with one stitch. A chest tube is placed in the pleura, and negative pressure is applied to depressurize the right pleura. The procedure is then repeated on the left side. The procedure for the left side is almost identical to that for the right side. We suggested that the left lowest port be located more posteriorly than the right one to ensure safe left atrial appendage exclusion [23,24]. The vital signs should be monitored carefully during carbon dioxide insufflation because the remnant positive pressure in the right pleura can cause temporary cardiac tamponade. Desaturation often occurs if the right lung is not re-expanded fully. The pericardium is opened a few centimeters posterior to the phrenic neve. Before pulmonary vein and ganglionated plexus ablation, the ligament of Marshall is dissected and ablated. Unlike on the right side, dissection inferior to the left lower pulmonary vein is not necessary to enter the oblique sinus. As on the right side, a rubber band is placed posterior to the left pulmonary vein antrum and is used to guide the bipolar clamp into place to ablate the left pulmonary vein antrum. When all ablations are complete and the conduction block is confirmed, the left atrial appendage is removed with an endoscopic stapling device or is excluded with AtriClip (AtriCure, Inc., Cincinnati, OH, USA). Pericardial closure is not usually necessary. A chest tube is placed in the pleura, and negative pressure is applied to depressurize the left pleura. If the patients are not in sinus rhythm by the end of the procedure, they are given a synchronized direct-current shock to establish sinus rhythm.

### 2.3. Conduction Block Test

Pulmonary vein isolation is confirmed via exit and entrance block tests using a flexible AtriCure Cooltip pen and Lab system (Atricure, Inc., Cincinnati, OH, USA) usually after initial delivery of energy for a total of six times. In this study, however, the clamp was released after each pulmonary vein clamping ablation, and a conduction block test was performed (Figure 1B,C). In other words, the conduction block test was not performed while the pulmonary vein atrium remained clamped. The minimum number of ablation lines required to achieve an electrical block was recorded. The exit block test was defined as the absence of conduction from the pulmonary veins to the left atrium, and the entrance block was defined as conduction from the left atrium to the pulmonary veins.

### 2.4. Postoperative Management and Follow-Up

The patients were monitored in the intensive care unit until the next day. A novel oral anticoagulant was prescribed from the first postoperative day. Rhythms of patients were monitored with telemetry and standard 12-lead electrocardiogram recordings until discharge. Patients were discharged with antiarrhythmics and an anticoagulant and were then followed up at 2 weeks, 3 months, and 6 months postoperatively. Echocardiography and 24-h Holter electrocardiogram monitoring were scheduled, usually between 3 and 6 months postoperatively.

### 2.5. Statistical Analysis

The perioperative data and follow-up data were obtained from the hospital records. Continuous variables were expressed as means and standard deviations when continuous variables were normally distributed. The median and interquartile range (IQR) between the 25th and 75th percentile values were described for some variables if the distribution of the data was skewed. The Kaplan–Meier method was used to estimate atrial tachycardia free survival rates. Categorical data were given as percentages. Statistical Package for the Social Sciences software, version 20.0 (IBM Corp, Armonk, NY, USA), was used for statistical data analysis.

## 3. Results

### 3.1. Patient Characteristics

This study included a total of 20 patients. Table 1 presents the preoperative characteristics. There were 19 men and one woman, and the median age was 61 years old (IQR 52.50–62.25 years). The median duration of AF was 78 months ([IQR 28.50–123.00 months), and 19 patients (95%) had long-standing persistent AF. Three patients (15%) had undergone prior radiofrequency catheter ablations. The median ejection fraction was 58% (IQR 56.50–61.00%), and the left atrial diameter was 46.05 mm (IQR 29.55–53.05 mm).

### 3.2. Operative Data

**Figure 2:**
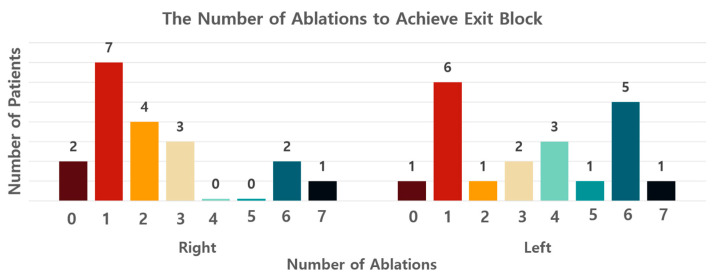
Number of pulmonary vein antral ablation lines to achieve an exit block is depicted as a graph. The block was achieved with the first ablation line in even patients with the right-side procedure and in six patients with the left side procedure.

Table 2 lists the operative data. The median surgery time was 135 min (IQR 131.00–152.25 min). The total number of ablations on each side of the procedure did not exceed seven owing to the possibility of pulmonary vein stenosis. Thus, the total number of ablations on each side was six or seven. Two patients who had a previous history of catheter ablation were found to have conduction block at the initial test before the first ablation. Three patients had unidirectional exit blocks: one on the left, one on the right, and one on both sides. The median number of ablation lines to achieve an electrical block, especially the exit block, was two on the right side and 3.5 on the left side. The number of ablations for each patient is depicted in Figure 2. The block was achieved just after the first ablation in seven patients on the right side and six patients on the left side. A median of six ablation lines around each pulmonary vein antrum were performed according to our protocol, even after the conduction block was verified. The mean size of the clip for left atrial appendage exclusion was 40 ± 3.35 mm.

### 3.3. Postoperative Data

**Figure 3:**
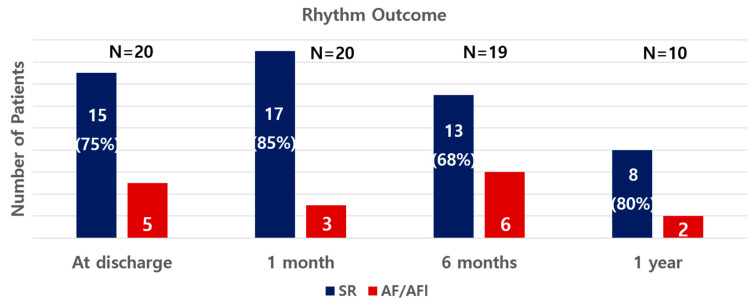
Rhythm outcome. Out of the total, 75% of patients were in sinus rhythm at discharge, 85% at 1 month, 68% at 6 months, and 80% at 1 year. AF; atrial fibrillation, AFl; atrial flutter, SR; sinus rhythm.

**Figure 4:**
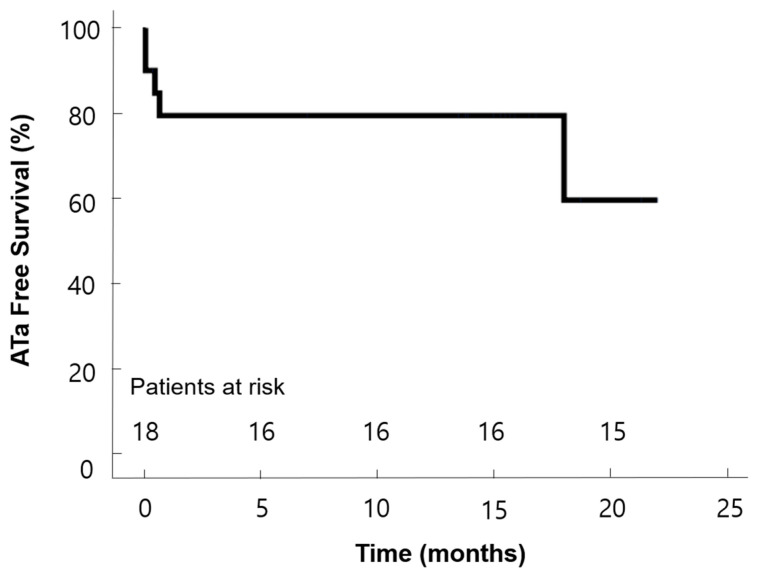
Kaplan–Meier curve of freedom from atrial tachyarrhythmia (ATa).

Table 3 presents the postoperative data. The median postoperative hospital stay was 5 days (IQR 4, 5 days). There were no reoperations, and no patients had postoperative pneumonia. Eight patients (40%) had undergone cardioversions before discharge. The rhythm outcomes are described in Figure 3. Fifteen patients (75%) were in sinus rhythm during discharge. The patients were evaluated with 24-h Holter at 6 months postoperatively. Thirteen of 19 patients (68%) were free from AF or atrial arrhythmia, and two patients experienced paroxysmal AF. One patient was found to have AF at the 6-month follow-up, but the rhythm was converted back to sinus rhythm after cardioversion at the 1-year follow-up. In 1 year, 17 patients were followed up, and 15 of them had sinus rhythm. Overall, during the follow-up period of 15.2 ± 1.3 months, 75% of patients were in sinus rhythm at the last follow-up. The atrial tachyarrhythmia-free survival rate was analyzed with the last cardiac rhythms. The rates were 79.4% at 10 months and 59.6% at 20 months, respectively (Figure 4). In 6 months, the echocardiography data of 18 patients showed no significant changes from baselines. There was one late complication with insignificant pulmonary vein stenosis. Postoperative computed tomography scans were taken in 11 patients at 11.4 ± 0.8 months. Minimal stenosis at the ostium of the left inferior pulmonary vein was identified in an asymptomatic patient who had been in sinus rhythm since the operation until 21.9 months postoperatively.

## 4. Discussion

AF is a common supraventricular arrhythmia characterized electrocardiographically by low-amplitude baseline oscillations (fibrillatory or f waves) and an irregularly irregular ventricular rhythm. The f waves have a rate of 300–600 beats/min and vary in amplitude, shape, and timing. AF often shows no definite symptoms. However, it can result in significant complications, such as thromboembolism [25]. The treatments for converting AF to sinus rhythm include cardioversion, pharmacologic control, catheter ablation, and surgical treatment. Among them, catheter ablation and surgical treatment are fundamental treatments in that they electrically isolate or eliminate arrhythmic foci. Strategies for eliminating AF involve anatomy- and electrophysiology-guided approaches [26]. The anatomical approach has developed along with surgical treatment for AF, while the electrophysiological approach has developed with advances in electrophysiology. To understand the mechanism of TTA ablation that we performed, it is necessary to first understand the development of surgical treatments and radiofrequency ablation techniques for AF. The left atrial isolation procedure, Corridor procedure, and atrial transection procedure were employed in the late 1980s, but they had certain limitations [27,28,29,30,31]. The Cox-maze procedure was eventually developed to address their limitations. Anatomical lesion sets have been established, as the Cox-maze procedure has undergone further modifications (from I to III). As a result, lesion sets of the Cox-maze III procedure have become the standard over the last 30 years. Prasad et al. reported a symptomatic AF-free survival rate 96.6% at 5.4 years without any difference between the stand-alone and concomitant operation groups [5]. Gomes et al. recently reported sinus rhythm maintenance rates of 88%, 85.1%, and 80.6% at 6, 24, and 36 months, respectively, after the Cox-maze III procedure [4]. However, the Cox-maze procedure is invasive because it requires sternotomy, cardiopulmonary bypass, and cardiac arrest by cardioplegia. Because of its invasive nature, stand-alone surgical AF treatment has been performed less frequently than concomitant operations for AF with other cardiac operations [5,6]. To reduce its invasiveness, various surgical modifications have been developed for stand-alone AF [32]. As radiofrequency ablation has developed, epicardial AF surgery without cardiopulmonary bypass has developed simultaneously because radiofrequency ablation can be applied epicardially. Radiofrequency radiation passes through tissues, and then resistive heating occurs within a narrow rim of tissue in direct contact with the electrode. Passive conduction of the heat continues from this interface to create lesions in the deeper tissues. Lesion transmurality has improved further with the introduction of bipolar radiofrequency epicardial ablation, rather than monopolar radiofrequency epicardial ablation. In particular, clamping bipolar ablation has made transmurality superior to linear monopolar ablation [12,33]. The device creates an algorithm to detect real-time measurement of lesion transmurality. The conductance between electrodes is measured during the ablation. The conductance being dropped to a stable minimum level is well correlated experimentally and clinically with histological transmural lesions [8,33,34,35,36,37]. Furthermore, a bipolar linear probe with two electrodes embedded side by side has been under development for the last 10 years in an effort to achieve better transmurality than with the monopolar device [35,38,39,40,41,42,43]. The bipolar linear and clamping probes have made the box-lesion more complete. Nowadays, all the introduced techniques are commercialized for epicardial ablation. In particular, with the use of thoracoscopy without sternotomy, epicardial ablation is possible while the heart beats. Therefore, thoracoscopic epicardial ablation is expected to further decrease the incidence of complications compared to the classic Cox-maze procedures [7]. However, the procedural details have not yet been established. For example, most previous studies of TTA have reported lesion sets [22,44,45,46,47]. Now, TTA can create almost all lesions that can be created via the Cox-maze procedure III except the cavotricuspid isthmus line and mitral isthmus line, which cannot be approached epicardially [48,49,50,51]. However, as far as we know, no previous study has reported the optimal number of ablation lines required around the pulmonary vein antrum. This study was intended to share our experiences with epicardial conduction block tests right after pulmonary vein antral clampings for ablation.

Optimum ablation can be defined as ablation that assures both conduction block and safe outcomes with a minimum number of ablation lines. The intraoperative conduction block can be confirmed by entrance and exit block tests, but some complex issues arise. During TTA, the conduction block is validated only by the epicardial test. The epicardial tissue might be injured and edematous immediately after ablation, which could lead to a false-positive epicardial block. Although intraoperative epicardial validation had been reported to have comparable results to the endocardial test [52], remnant pulmonary vein potentials have been found in 19% of patients in electrophysiologic studies performed 5 days after TTA [53]. This finding suggests that more ablation lines might be required, even after validation of the intraoperative conduction block.

However, more ablation lines could lead to more complications, such as pulmonary vein stenosis. De Greef et al. [20] reported permanent pulmonary vein stenosis in only 0.003% of cases after catheter ablations. On the other hand, Samuel et al. [54] conducted a multicenter, single-blind, randomized, controlled trial to determine the incidence of pulmonary vein stenosis after catheter ablation. When they performed 197 computed tomographic or magnetic resonance angiographies, pulmonary vein stenosis was identified in 20.8% of patients and 8.2% of ablated pulmonary veins. Although it is uncertain whether mild-to-moderate pulmonary vein stenosis is noteworthy, stenosis may progress, and long-term outcomes should be closely monitored. Surgeons should be aware of complications and should be careful when raising the number of ablation lines. In our study, postoperative chest computed tomography was performed in 11 patients. Ten patients did not have definite pulmonary vein stenosis; only one patient was diagnosed with minimal stenosis of the ostium in the left inferior pulmonary vein. For this patient, we additionally ablated the left pulmonary vein antrum during surgery because we detected arrhythmic focus in the left pulmonary vein carina after the routine pulmonary vein isolation. His cardiac rhythm was constantly in sinus rhythm until 21.8 months after surgery, and he has complained of no special symptoms thus far. The structure of the AtriCure Isolator Transpolar Clamp (Atricure, Inc., Cincinnati, OH, USA) that we used has a curvature to clamp the pulmonary vein antrum and not the pulmonary veins themselves. We believe that this fact is why there are separate clamps for left and right use. As described in the Materials and Methods section, correct positioning of the clamp on the pulmonary vein antrum is possible with the AtriCure Isolator Transpolar Clamp (Atricure, Inc., Cincinnati, OH, USA).

Previous studies of TTA have revealed that surgeons usually create two to 10 pulmonary vein antral circumferential ablation lines with the Gemini-s ablation device (Medtronic, Minneapolis, MN, USA) or AtriCure Isolator Transpolar Clamp (Atricure, Inc., Cincinnati, OH, USA) [17,18,19,22,55]. Yilmaz et al. [17] created at least three lines; Gaynor et al. [56], two lines; Zotov et al. [19], 10 lines; Wolf et al. [22], at least two lines; Kwon et al. [21], six lines; and Guo et al. [55], three to five lines.

The additional lines might be created according to the conduction block test, but there are no data on additional procedures. The results of the current study showed that the block was achieved in 35% of patients, even with the first ablation. Additionally, 84% and 50% of patients achieved conduction block within three ablations on the right and left side, respectively. The conduction block was mostly achieved within seven ablations in the current study with 20 patients. Based on the results and the possibility of a false-positive conduction block test result, at least three pulmonary vein ablation lines should be considered.

This study has several limitations. First, it was based on experiences at a single center with a small sample size. It was cumbersome to insert and remove the clamping device repeatedly for conduction block testing every time the pulmonary vein antrum was ablated. The cumbersome procedures of additional repetitive conduction block tests limited the sample size to be even smaller. Second, the follow-up period, which was limited to 15.2 ± 1.3 months, was relatively short,. Third, there is a possibility that the intraoperative conduction block was a false positive, which may not guarantee long-term results. The primary endpoint of our study was the number of ablation lines required to achieve conduction block intraoperatively, not the postoperative rhythm outcomes because the postoperative rhythm outcomes were inappropriate to evaluate the minimum number of ablation lines, owing to many other variables, such as other foci of AF, medications, and patient factors. Further studies are required to determine the correlation between conduction block and rhythm outcomes.

## 5. Conclusions

The results of this study suggested that, in the majority of cases, the pulmonary vein conduction block was achieved with two to 3.5 ablation lines around each pulmonary vein antrum. We hope that this study may provide evidence to reduce the number of unnecessary ablation lines in the future.

## Figures and Tables

**Table 1:** Baseline Characteristics.

Age, years	58.70 ± 10.17
Female, n (%)	1 (5)
AF duration, months	88.15 ± 70.70
Long-standing AF, n (%)	19 (95)
Prior RFCA	3 (15)
Hypertension, n (%)	8 (40)
Diabetes mellitus, n (%)	4 (20)
Congestive heart failure, n (%)	6 (30)
Prior stroke, n (%)	4 (20)
Thyroid disease, n (%)	3 (15)
**Echocardiographic parameters**	
Left ventricular ejection fraction (%)	56.63 ± 8.37
Left atrial diameter (mm)	46.47 ± 7.95
Left atrial volume index (mL/min)	53.92 ± 17.97

AF, atrial fibrillation; RFCA, radiofrequency catheter ablation

**Table 2:** Operative Data.

Operative Data	
Operative time, minutes	140.25 ± 16.02
**Right-side procedures**	
Total number of ablations	6.3 ± 0.47
Confirmed PVI, n (%)	18 (90)
Entrance block, n (%)	18 (90)
Exit block, n (%)	19 (90)
Exit block, the minimum number of ablations (median, IQR)	2 (1, 3)
**Left side procedures**	
Total number of ablations	6.35 ± 0.49
Confirmed PVI, n (%)	18 (90)
Entrance block, n (%)	18 (90)
Exit block, n (%)	20 (100)
Exit block, the minimum number of ablations (median, IQR)	3.5 (1, 6)
LA appendage exclusion, size, mm	40 ± 3.35

PVI, pulmonary vein isolation; LA; left atrial, IQR; interquartile range

**Table 3:** Postoperative Data.

Postoperative Data	
Postoperative hospital stay, days	6.4 ± 1.8
**Complication**	
Reintubation, number of patients, n (%)	0 (0)
Reoperation, number of patients, n (%)	0 (0)
Incidence of pneumonia, n (%)	0 (0)
**Rhythm**	
Cardioversion during admission, n (%)	8 (40)
Sinus rhythm at discharge, n (%)	15 (75)
Sinus rhythm at 6 months, n (%)	13 (68)
Sinus rhythm at the last follow-up, n (%)	15 (75)
**Echocardiographic parameters at 6 months**	
Left ventricular ejection fraction (%)	57.72 ± 6.53
Left atrial diameter (mm)	48.42 ± 7.11
Left atrial volume index (mL/min)	46.04 ± 13.29

## Data Availability

The data presented in this study are available on request from the corresponding author.
